# Supplementary material for: Multiscale reconfiguration induced highly saturated poling in lead-free piezoceramics for giant energy conversion
Source: Nat Commun. 2024 Mar 22;15:2560. doi: 10.1038/s41467-024-46894-5 (PMC10959963; doi:10.1038/s41467-024-46894-5)
Supplement: Supplementary file 1 — Supplementary Information [file 41467_2024_46894_MOESM1_ESM.pdf]

# Supplementary Information

## Multiscale Reconfiguration Induced Highly Saturated Poling in Lead-Free Piezoceramics for Giant Energy Conversion

*Jinfeng Lin[1], Jin Qian[1], Guanglong Ge[1], Yuxuan Yang[2], Jiangfan Li[3], Xiao Wu[4], Guohui Li[1], Simin Wang[1], Yingchun Liu[5], Jialiang Zhang[3], Jiwei Zhai[1,✉], Xiaoming Shi[6,✉], Haijun Wu[2,✉]*

[1]School of Materials Science and Engineering, Tongji University, Shanghai 201804, China. [2]State Key Laboratory for Mechanical Behavior of Materials, Xi'an Jiaotong University, Xi'an 710049, P. R. China. [3]School of Physics, State Key Laboratory of Crystal Materials, Shandong University, Jinan 250100, China. [4]Key Laboratory of Eco-materials Advanced Technology, College of Materials Science and Engineering, Fuzhou University, Fuzhou 350108, China. [5]Functional Materials and Acoustooptic Instruments Institute, Harbin Institute of Technology, Harbin 150080, China. [6]Department of Physics, University of Science and Technology Beijing, Beijing 100083, China. ✉ E-mail: apzhai@tongji.edu.cn (J. Zhai); shiming_870@163.com (X. Shi); wuhaijunnavy@xjtu.edu.cn (H. Wu);

**This file includes:**

Supplementary Methods

Supplementary Figures S1 to S21

Supplementary References

**Supplementary Methods**

**Sample Preparation:** A series of $(K_{0.505}Na_{0.5(0.99-x\%)}Ca_{0.01}Bi_{0.5\cdot x\%})(Nb_{0.965(0.99-x\%)}Sb_{0.035(0.99-x\%)}Zr_{0.01}Hf_{0.98\cdot x\%}Ti_{0.02\cdot x\%})O_3$ ($x$ = 0 - 7, abbreviated as $x$BHT) lead-free piezoceramic powders were designed and fabricated by using the conventional solid-state method. All high purity raw materials, including $K_2CO_3$ (Aladdin, 99.5%), $Na_2CO_3$ (Aladdin, 99.8%), $Nb_2O_5$ (Aladdin, 99.98%), $CaCO_3$ (Sinopharm, 99.99%), $Bi_2O_3$ (Alfa Aesar, 99.975%), $HfO_2$ (Aladdin, 99.99%), $ZrO_2$ (Aladdin, 99.99%), $TiO_2$ (Sinopharm, 99.8%), and $Sb_2O_3$ (Alfa Aesar, 99.9%) were ball milled with ethyl alcohol and $ZrO_2$ balls after weighting according to the formula. Prior to secondary ball milling, the dried powder from primary ball milling needs to be calcined at 845-850 °C for 5 h. For the random ceramics (denote as R-$x$BHT), the dried calcined powers were sieved and pressed into pellets of 12-mm diameter and 1-mm thickness under the pressure of 200-300 MPa with a binder of 6 wt% polyvinyl alcohol. For the textured ceramics (denote as T-$x$BHT), the template grain growth (TGG) technology based on tape casting process is required (Fig. S1c). The ceramic slurry was synthesized by mixing the dried calcined powers with ethanol/toluene co-solvents, organic binders and 3 wt% high-quality NN templates (Fig. S4d). After roller milling for 8 h, the homogeneous slurry was casted by using casting machine. Next, the dried tapes were sequentially cut, laminated and pressed into pellets under 200-300 MPa pressure at 60 °C for 10 min. Finally, all the pellets for both random and texture ones were heated to 600 °C at 1 °C/min to burn out the binder and then sintered at 1180-1210 °C for 6-8 h in air by a two-step sintering, which was described in our previous report.

**Fabrication of Energy Converters:**

**Energy Harvester:** The ceramic was prepared into a piezoelectric circular diaphragm (PCD) energy harvester for experimental study (Figs. 6a and S20a, b). Firstly, the ceramic was prepared and sintered into a ceramic with a diameter of ~ 22 mm. Next, the sintered ceramic was thinned to a thickness of ~ 0.22 mm, coated with 20 mm diameter silver electrodes on both sides, and annealed at 560 °C for 10 minutes. After being fully poled at 20 kV cm$^{-1}$ by AC

electric field, the poled ceramic, proof mass (14 g), wires and studdle were glued to the brass substrate and cured to form a PCD energy harvester, which was then mounted on the shaker for testing. The vibration frequency and amplitude were controlled by emitting signals from a signal generator (33220A, Agilent). The vibration signal was amplified by the power amplifier (YE5871A) and transmitted to the mechanical shaker (JCK-5), capable of providing the vibration acceleration. The acceleration of the shaker was fixed to 2 g (g = 9.8 m/s$^2$). A digital oscilloscope records the real-time voltages of the resistance.

**Ultrasonic Transducer:** The ceramics was prepared into an ultrasonic transducers for experimental study (Figs. 6j and S21). Firstly, the ceramics was prepared and sintered into a thick ceramic with an area of ~ 12 mm × 12mm. Next, the sintered ceramic was thinned to a thickness of ~ 1 mm, coated with silver or gold electrodes on both sides. After being fully poled at 20 kV cm$^{-1}$ by AC electric field, the ultrasonic transducer as shown in Figs. 6j and S21 was prepared using tungsten powders mixed into epoxy resin (E51) as backing material, and alumina powders mixed into epoxy resin (E51) as matching layers.

**Characterization of Electrical Properties:** After mechanically thinning to 0.3-0.7 mm thickness, xBHT ceramic samples were coated by Ag electrodes on both sides for dielectric, ferroelectric, and electric field-induced strain tests. Polarization (*P-E*) and strain (*S-E*) hysteresis loops were measured using a ferroelectric test system (Precision Premier II) at 10 Hz. After poled at 30 kV cm$^{-1}$ by AC electric field at room temperature, the piezoelectric coefficient ($d_{33}$) was tested at room temperature using a quasi-static $d_{33}$ meter (ZJ-6A, Institute of Acoustics, China). The temperature-dependent dielectric constants were achieved by the Agilent E4980A LCR meter and the Keithley 2410 Source Meter equipped with a heating stage (-100-360 °C). The planar electromechanical coupling factor $k_p$ was determined using an impedance analyzer (HP4294A, Agilent, USA).

**Ex-situ structural characterization:** Acid-etched domain structures were observed on a scanning electron microscopy (SEM) JSM-7610F. Before acid-etching, to reduce the impact of

temperature on the poled sample, the required hot melt adhesive operation temperature for polishing is controlled at around 100 °C or as low as possible. A mixed aqueous solution of HCl acid (with the mass concentration of about 37%, Laiyang Kangde Chem. Co., Ltd.) and HF acid (with a mass concentration ≥ 40%, Tianjin Kemiou Chemical Reagent Co., Ltd.) in a volume ratio of 1:1 was used the etchant. After experimental confirmation, the optimal time for domain chemical corrosion of the *x*BHT ceramics is ~ 2 minutes 15-30 s. The surface and cross-section microstructure of the ceramics were investigated with a scanning electron microscope (SEM, HITACHI, TM4000Plus). Electron backscatter diffraction (EBSD) pole figure and inverse pole figure for texture degree analyses were obtained by a field emission scanning electron microscope (FEI Magellan 400). The distribution of stress/strain distribution of PCD energy harvester is simulated using COMSOL Multiphysics. The specimens for transmission electron microscopy (TEM) and atomic-resolution scanning transmission electron microscopy (STEM) were prepared by mechanical thinning, ultrasonic cutting, and Ar-ion thinning until electrons can penetrate the samples with a thickness of ~ 30-50 nm. To reduce the impact of temperature on the poled sample, not only the required hot melt adhesive operation temperature for mechanical thinning is controlled at around 100 °C or as low as possible, but also liquid nitrogen is assisted in the Ar-ion thinning process. A JEOL JEM-2100F microscope was used to acquire the bright-field images for domain morphology. The atomic-scale imaging was carried out on a $C_s$-corrected Hitachi HF5000 microscope with ultra-high resolution (UHR) mode and a convergence/collection semi-angle of 20 mrad/60-320 mrad.

**Field-induced structural transition:** In-situ XRD under different temperatures were collected using X-ray diffractometer (PANalytical Empyrean, Holland). One self-made in-situ electric field XRD sample holder was used to test the phase structure at different applied voltages (0-800 V, sample thickness ~ 0.3 mm). The translucent gold electrodes by DC sputtering are used as electrodes for testing the in-situ variable electric field XRD. Using interdigital gold electrode, in-situ variable electric field local domain morphology of optimized/non-optimized vertical

piezoresponse force microscopy (OV or V-PFM) were obtained by piezo-response force microscope with additional voltage adding devices (PFM, Dimension Icon, Bruker, United States) (0-200 V, the width of the interdigital electrode is 100 μm). To avoid electrical signal interference, the voltage was temporarily removed before testing OV or V-PFM. In-situ electric field dielectric curves were achieved by Agilent E4980A LCR meter and the Keithley 2410 Source Meter equipped with additional voltage-adding devices (thickness of sample ~ 0.3 mm).

**Phase-field model:** To describe the domain structures in textured and random grain structures, an order parameter $\eta$ was employed to represent the different crystal orientations. Use two sets of coordinate systems to represent polarization in different grain structures. Within each grain, the domain structure can be described as local spontaneous polarization $\mathbf{P}_L$. In the global coordinate system, a global polarization **P** and a displacement field **u** were adopted as the order parameters in the phase-field model. The temporal evolution of the polarization is described by the time-dependent Ginzburg-Landau (TDGL) equation and the stress/electric field equilibrium equation[1],

$$\frac{\partial \mathbf{P}_i}{\partial t} = -L\frac{\delta F}{\delta \mathbf{P}_i} + \mathbf{E}_i^{thermal} \tag{1}$$

$$\frac{\partial}{\partial x_j}(\sigma_{ij}(r,t)) = 0 \tag{2}$$

$$\nabla \cdot \mathbf{D} = \rho_f \tag{3}$$

Here, $L$ is a kinetic coefficient related to domain wall mobility, $F$ is the total free energy of the system, $\frac{\delta F}{\delta \mathbf{P}_j}$ is the thermodynamic driving force, $\sigma_{\mathrm{ij}}$ is the stress tensor, **D** is the electric displacement, $\rho_f$ is the free charge density, $r$ and $t$ are the spatial coordinate and time, respectively. The total free energy of a bulk system can be defined as follows,

$$F = F_{Land}(\mathbf{P}) + F_{grad}(\mathbf{P}) + F_{elastic}(\mathbf{P}) + F_{elec}(\mathbf{P},\mathbf{E}) = \int_V (f_{Land} + f_{grad} + f_{elastic} + f_{elec})\, dV \tag{4}$$

Where $F$ includes the bulk free energy $F_{bulk}$(**P**), domain-wall energy $F_{grad}$(**P**), elastic energy

$F_{elastic}(\mathbf{P})$, and electrostatic energy $F_{elec}(\mathbf{P},\mathbf{E})$, $\mathbf{E}$ is the applied static electric field. $f_{\mathrm{Land}}$, $f_{\mathrm{grad}}$, $f_{\mathrm{elastic}}$ and $f_{\mathrm{elec}}$ are the corresponding energy density.

The bulk free energy density in a given grain can be expanded in terms of polarization components. For KNN system, the bulk free energy density can be described as:

$$\begin{aligned} f_{Land} &= a_1(P_{L1}^2+P_{L2}^2+P_{L3}^2)+a_{11}(P_{L1}^4+P_{L2}^4+P_{L3}^4)+a_{12}(P_{L1}^2P_{L2}^2+P_{L1}^2P_{L3}^2+P_{L2}^2P_{L3}^2)+a_{11}(P_{L1}^6+P_{L2}^6+P_{L3}^6) \\ &+a_{112}[P_{L1}^4(P_{L2}^2+P_{L3}^2)+P_{L3}^4(P_{L2}^2+P_{L1}^2)+P_{L2}^4(P_{L3}^2+P_{L1}^2)]+a_{123}P_{L1}^2P_{L2}^2P_{l3}^2+a_{1111}(P_{L1}^8+P_{L2}^8+P_{L3}^8) \\ &+a_{1112}[P_{L1}^6(P_{L2}^2+P_{L3}^2)+P_{L3}^6(P_{L2}^2+P_{L1}^2)+P_{L2}^6(P_{L3}^2+P_{L1}^2)]+a_{1122}(P_{L1}^4P_{L2}^4+P_{L1}^4P_{L3}^4+P_{L2}^4P_{L3}^4) \\ &+a_{1123}(P_{L1}^4P_{L2}^2P_{L3}^2+P_{L1}^2P_{L2}^4P_{L3}^2+P_{L1}^2P_{L2}^2P_{L3}^4) \end{aligned} \tag{5}$$

where $G$ is the Gibbs free energy of a cubic ferroelectric system, $a_1$-$a_{1123}$ is the landau coefficients and $\mathbf{P}_L$ is the polarization field in the local crystallographic coordinate system within each grain. A standard global coordinate system for all grains was introduced to solve the polycrystal's elasticity and electrostatic equilibrium equation. Three Euler angles was used to describe the orientation of different grain in the polycrystalline structure. Thus, the transformation matrix from the global to local coordinate system is given by:

$$tr = \begin{pmatrix} \cos\varphi\cos\psi-\cos\theta\sin\varphi\sin\psi & \sin\varphi\cos\psi+\cos\theta\cos\varphi\sin\psi & \sin\theta\sin\psi \\ -\cos\theta\cos\psi\sin\varphi-\cos\varphi\sin\psi & \cos\theta\cos\varphi\cos\psi-\sin\varphi\sin\psi & \sin\theta\cos\psi \\ \sin\theta\sin\varphi & -\cos\varphi\sin\theta & \cos\theta \end{pmatrix} \tag{6}$$

The polarization in the local coordinate system can be described as a transformation from the polarization in the global coordinate system through

$$\mathbf{P}_{Li} = \frac{1}{2} tr_{ij}\mathbf{P}_j \tag{7}$$

The gradient energy density in an anisotropic system can be calculated by

$$f_{grad} = \frac{1}{2} g_{ijkl}\mathbf{P}_{i,j}\mathbf{P}_{k,l} \tag{8}$$

where $g_{ijkl}$ is the gradient energy coefficient and $\mathbf{P}_{i,j} = \frac{\partial P_i}{\partial x_j}$ The elastic energy density can be described as:

$$f_{elas} = \frac{1}{2} c_{ijkl} e_{ij} e_{kl} = \frac{1}{2} c_{ijkl}(\varepsilon_{ij}-\varepsilon_{ij}^0)(\varepsilon_{kl}-\varepsilon_{kl}^0) \tag{9}$$

where $c_{ijkl}$ is the stiffness tensor, $e_{ij}$ is the elastic strain tensor, $\varepsilon_{ij}$ is the total strain tensor, and

$\varepsilon_{ij}^{0}$ is the eigenstrain,

$$\varepsilon_{ij}^{0} = \varepsilon_{Lij}^{0} = Q_{ijkl}\mathbf{P}_{Lk}\mathbf{P}_{Ll} \tag{10}$$

where $\varepsilon_{Lij}^{0}$ is the eigenstrain with respect to the local coordinate system, $Q_{ijkl}$ is the electrostrictive coefficient tensor. Therefore, the eigenstrain in the global coordinate system can be obtained from

$$\varepsilon_{ij}^{0} = tr_{ki}tr_{lj}\varepsilon_{Lkl}^{0} \tag{11}$$

The electrostatic energy density $f_{\mathrm{elec}}$ of the system in phase-field simulation is given by,

$$f_{elec} = -\mathbf{P}_i(r)(\mathbf{E}_i(r)+\mathbf{E}_{RF}) - \frac{1}{2}\mathbf{P}_i(r)\mathbf{E}_i^{in}(r) \tag{12}$$

where $\mathbf{E}_i^{in}(r)$ is the E-field induced by the dipole moments, $\mathbf{E}_i(r)$ is the applied elecric field and $\mathbf{E}_{RF}$ is the local electric field caused by the random point defects.

The parameters[2] (all in SI units) in details are $a_1 = 4.29(\,Coth\left[\frac{140}{T}\right] - Coth\left[\frac{140}{657}\right])\times 10^7$, $a_{11} = -2.73\times 10^8$, $a_{12} = 1.0861\times 10^9$, $a_{111} = 3.04\times 10^9$, $a_{112} = -2.73\times 10^9$, $a_{123} = 1.55\times 10^{10}$, $a_{1111} = 2.4\times 10^{10}$, $a_{1112} = 3.73\times 10^9$, $a_{1122} = 3.34\times 10^{10}$, $a_{123} = -6.2\times 10^{10}$, $Q_{11} = 0.13$, $Q_{12} = -0.047$, $Q_{44} = 0.052$, $s_{11} = 5.5\times 10^{-11}$, $s_{12} = -1.6\times 10^{-10}$, $s_{44} = 1.3\times 10^{-9}$. Where $s$ is the compliance coefficients and $Q$ is the electrostrictive coefficients. A random electric field that obeys the Gaussian distribution $N(0, \Delta)$ was applied, where $\Delta$ is the variance of the Gaussian distribution and can be connected with doping concentration. The simulation scale is 256 $dx$ × 256 $dz$. The grid scales $dx$ and $dz$ are 1 μm. 9 different grain orientations with the rotations about z direction from -45 to 45 degrees was used to account for the random grain structures. For the textured grain structure, the orientations were fixed with 45 degrees. Fourier method was used for solving the equations. The open circuit electrical boundary condition and the periodic mechanical boundary conditions are adopted in the calculations.

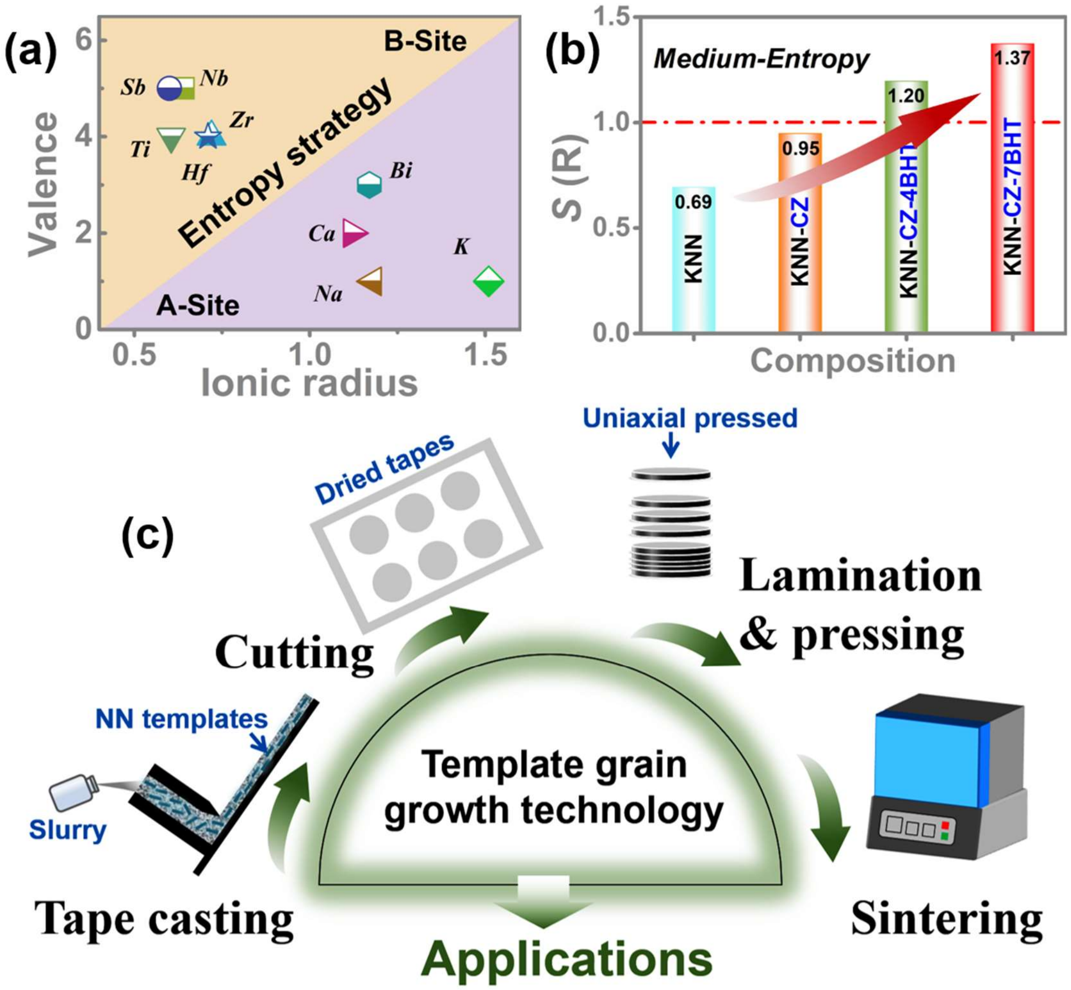


**Figure S1. Entropy modulation strategy and preparation process of the T-*x*BHT ceramics.** (a) The design strategy of medium-entropy for high-performance KNN-based piezoceramics. (b) The atomic configuration entropy $S_{\text{config}}$ of the $x$BHT ceramics. (c) Preparation process of the textured ceramics.

The construction of new phase boundary (NPB) requires both $T_{\text{R-O}}$ and $T_{\text{O-T}}$ to be moved to or near room temperature simultaneously. Thus, choosing the appropriate additives and tailoring their content are the most important factors for constructing the NPB. It was found that $Sb^{5+}$, $CaZrO_3$ and $Bi_{0.5}A_{0.5}BO_3$ (A = K/Na, B = Hf/Ti) all contribute to the decrease of $T_{\text{O-T}}$ and the increase of $T_{\text{R-O}}$. The difference is that both $Sb^{5+}$ and $CaZrO_3$ are more focused on the increase of $T_{\text{R-O}}$, while $Bi_{0.5}A_{0.5}BO_3$ (A = K/Na, B = Hf/Ti) is more focused on the increase of $T_{\text{O-T}}$.[3,4] Therefore, the involved Sb, Zr, Hf, Ti, Ca and Bi elements are utilized, which takes into account not only the modulation of atomic configuration entropy $S_{\text{config}}$, but also their respective ability to move the phase boundaries.

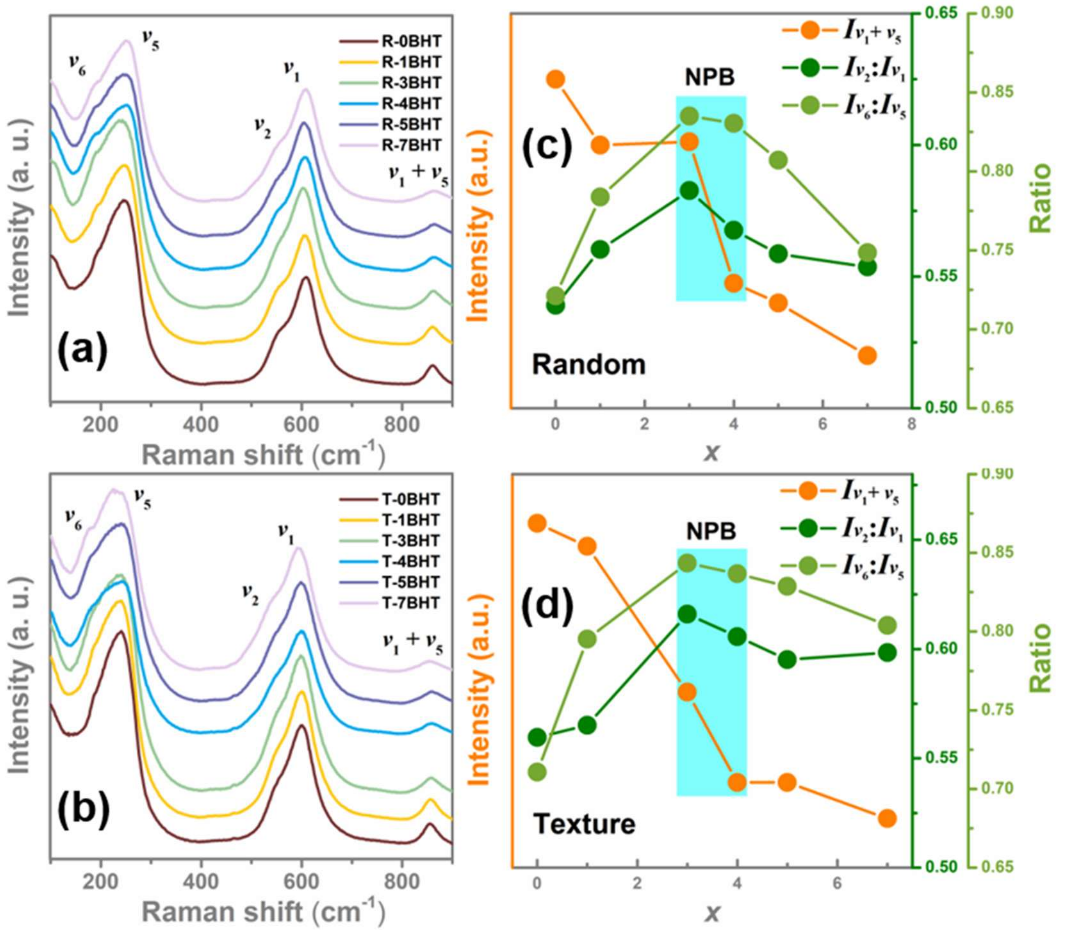


**Figure S2. Analysis of Raman spectra.** Raman spectra for the (a) random and (b) textured *x*BHT ceramics. Raman shift of different modes for the (c) random and (d) textured *x*BHT ceramics.

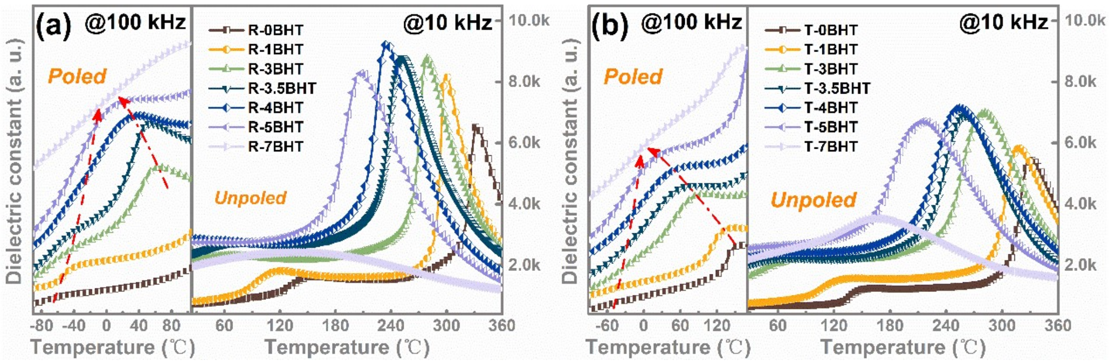


**Figure S3. Temperature-dependent dielectric constants.** The temperature-dependent dielectric constants for the (a) random and (b) textured *x*BHT ceramics from liquid nitrogen temperature to high temperature.

The reason of suppressing dielectric constant by textured engineering is due to two dominant factors.[5] The first is the elastoelectric composite effect, caused by interfacial stresses due to the lattice mismatch between KNN matrix and the NN templates. The second is associated with the discrepancy in electrical properties. Because the introduced NN templates has a lower dielectric constant than *x*BHT matrix, thus suppressing or even lowering the dielectric constant of the T-*x*BHT ceramics.

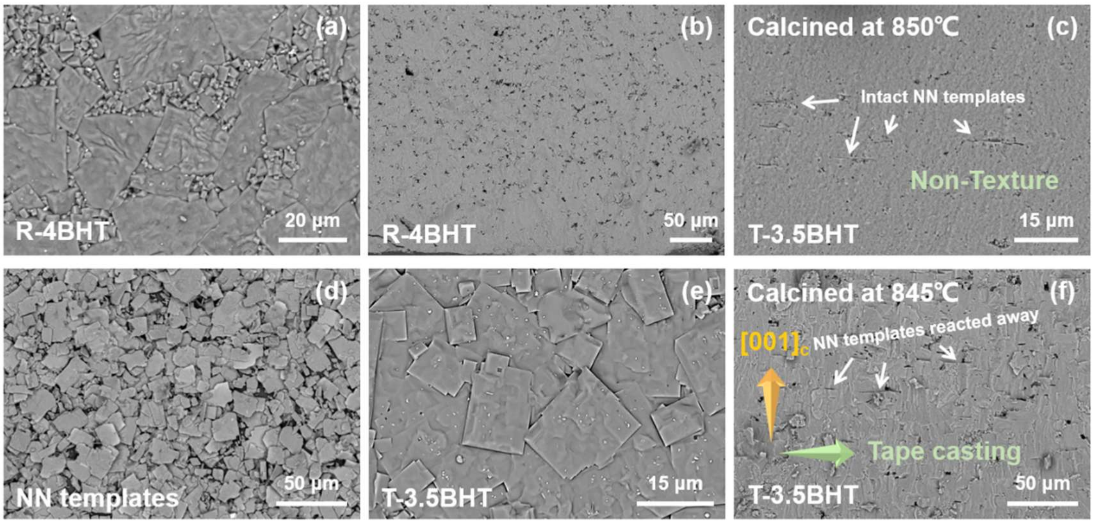


**Figure S4. Analysis of scanning electron microscope (SEM).** The surface (a) and cross-sectional (b) morphology of the R-4BHT ceramics. (c) The cross-sectional morphology of the T-3.5BHT ceramics calcined at 850 ℃. (d) The morphology of the NN templates. The surface (e) and cross-sectional (f) morphology of the T-3.5BHT ceramics calcined at 845 ℃.

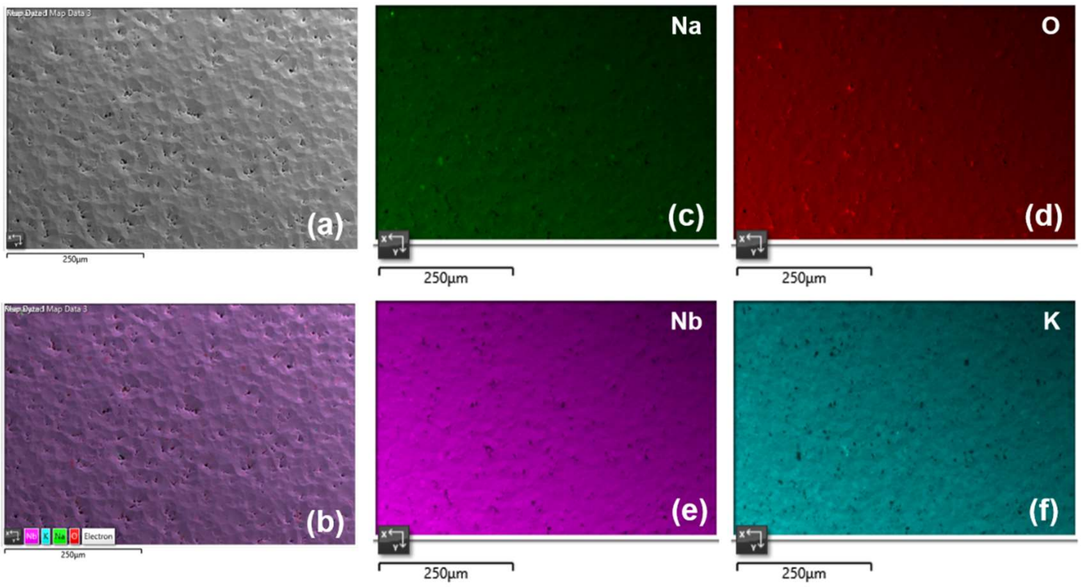


**Figure S5. Energy dispersive spectroscopy (EDS) element maps.** (a) SEM image of the surface of the polished T-3.5BHT ceramics. (b) Overlay chart of the EDS maps with different elements. The EDS maps of (c) Na, (d) O, (e) Nb and (f) K, respectively.

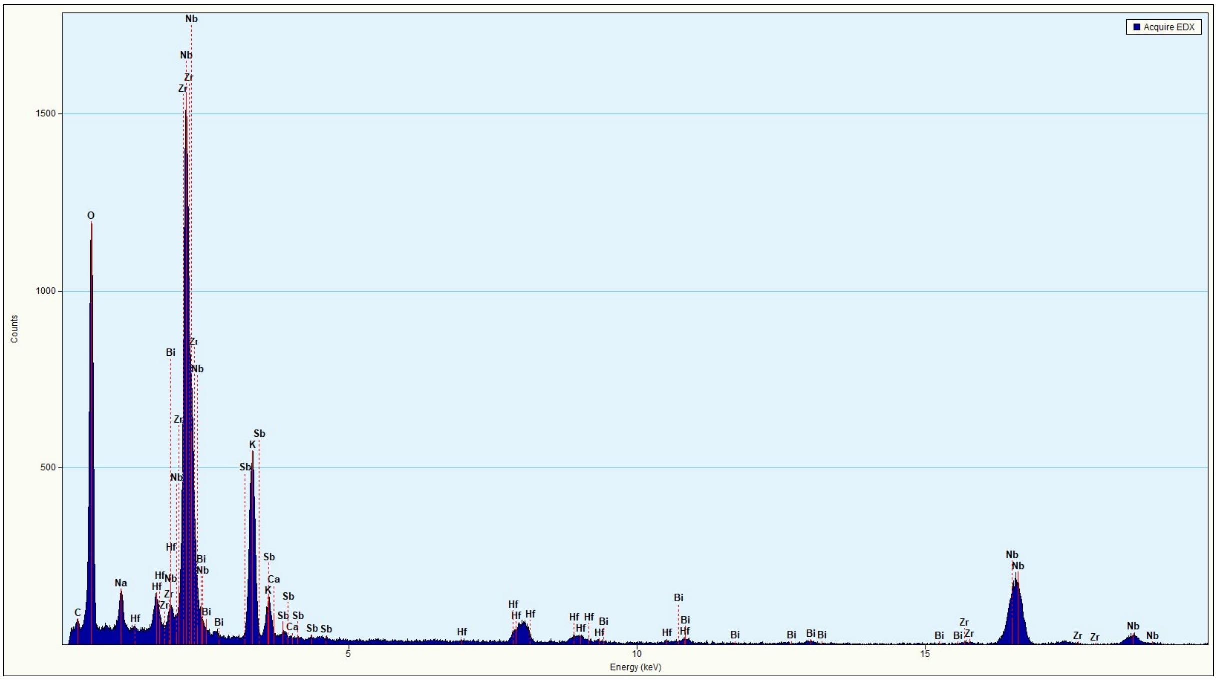


**Figure S6. Energy dispersive spectroscopy (EDS) point analysis.** EDS point analysis of microregion based on transmission electron microscope (TEM) of the T-3.5BHT ceramics.

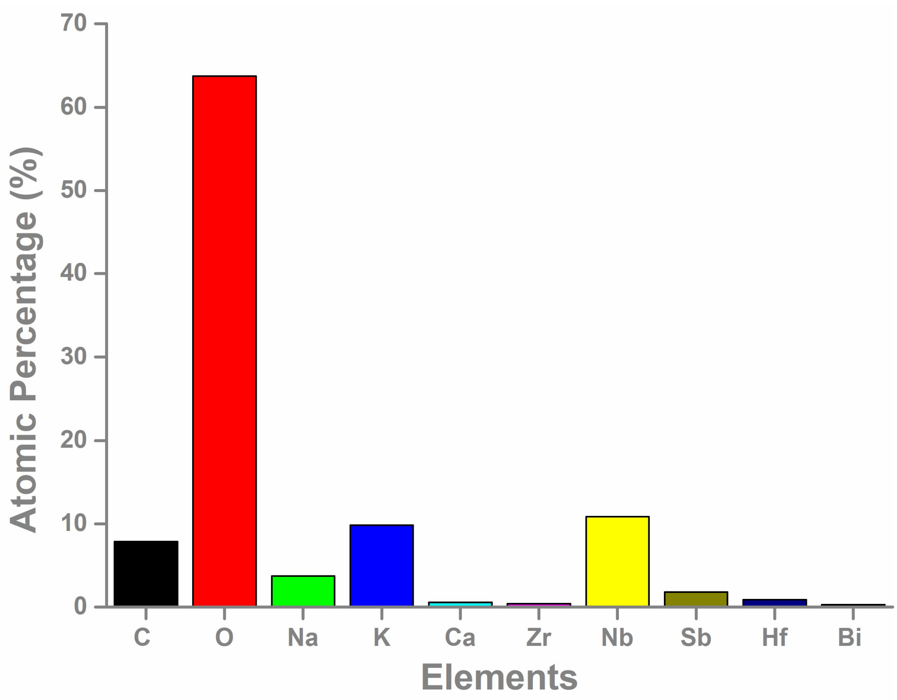


**Figure S7. Element content.** Atomic percentage of microregions based on EDS point analysis via TEM of the T-3.5BHT ceramics.

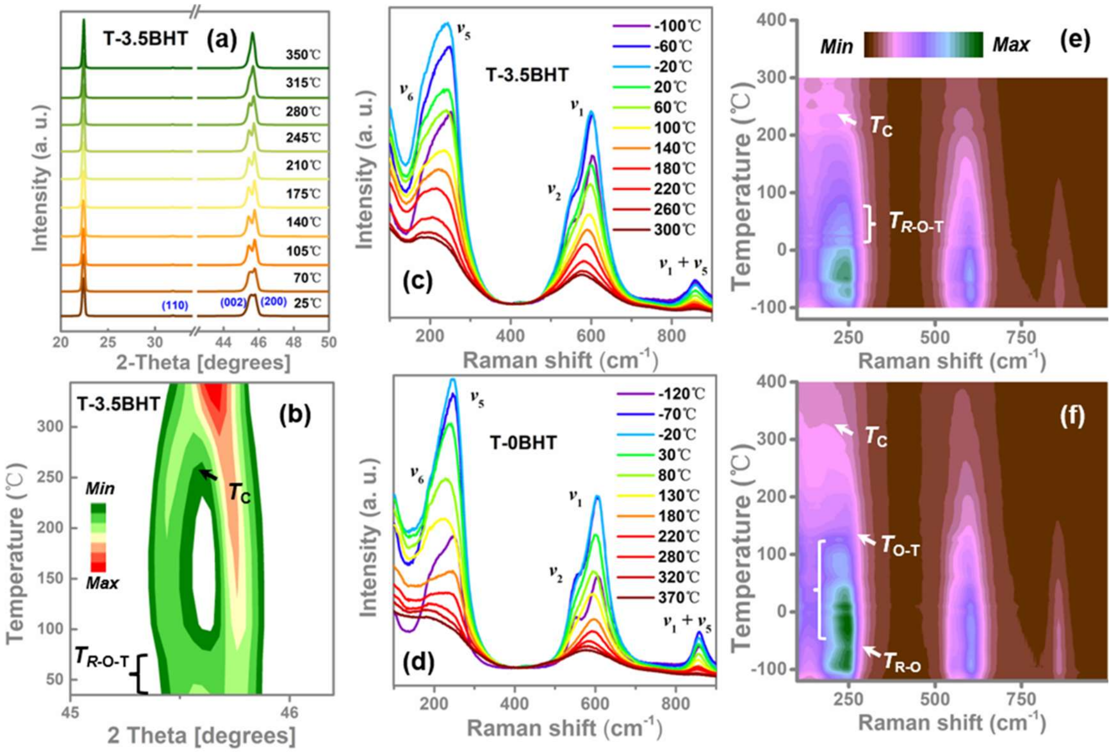


**Figure S8. Temperature-dependent XRD and Raman spectra analysis.** Stack lines (a) and two-dimensional map (b) of in-situ XRD under different temperatures for the T-3.5BHT ceramics. Stack lines (c) and two-dimensional map (e) of In-situ Raman spectra under different temperatures for the T-3.5BHT ceramics. Stack lines (d) and two-dimensional map (f) of In-situ Raman spectra under different temperatures for T-0BHT ceramics.

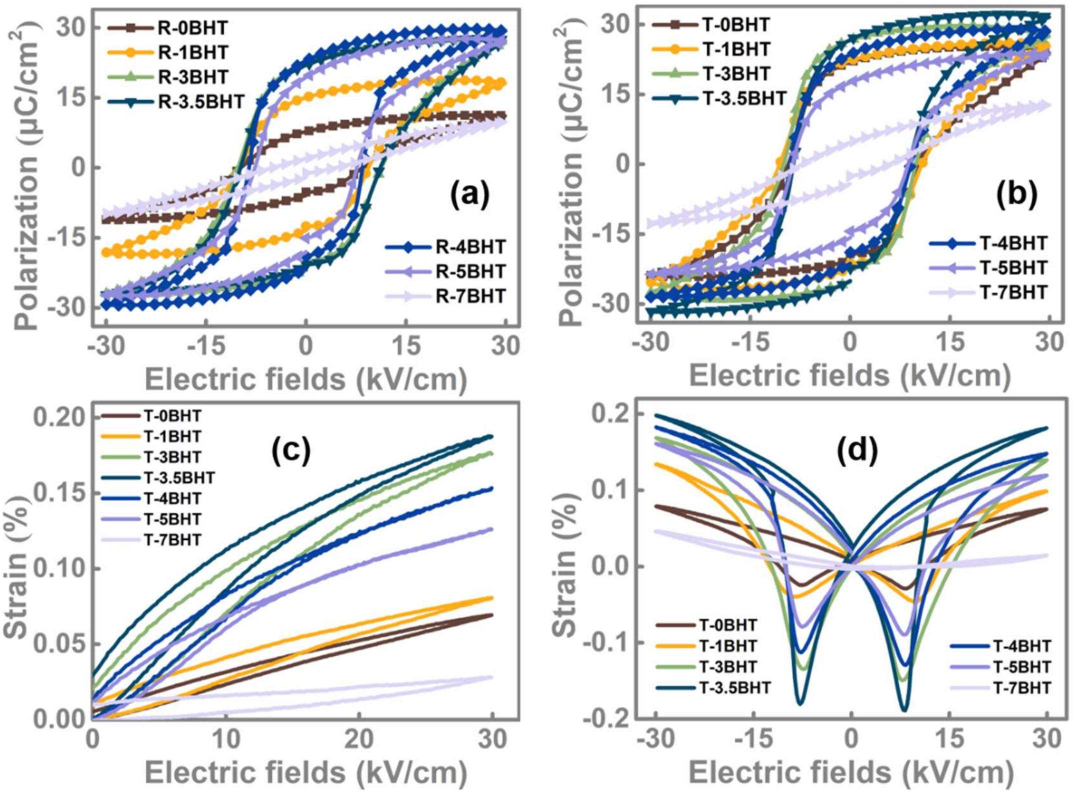


**Figure S9. *P-E* hysteresis loops and strain-electric field curves.** *P-E* hysteresis loops for the (a) random and (b) textured *x*BHT ceramics. c) Unipolar and d) bipolar strain-electric field (*S-E*) curves for T-*x*BHT ceramics.

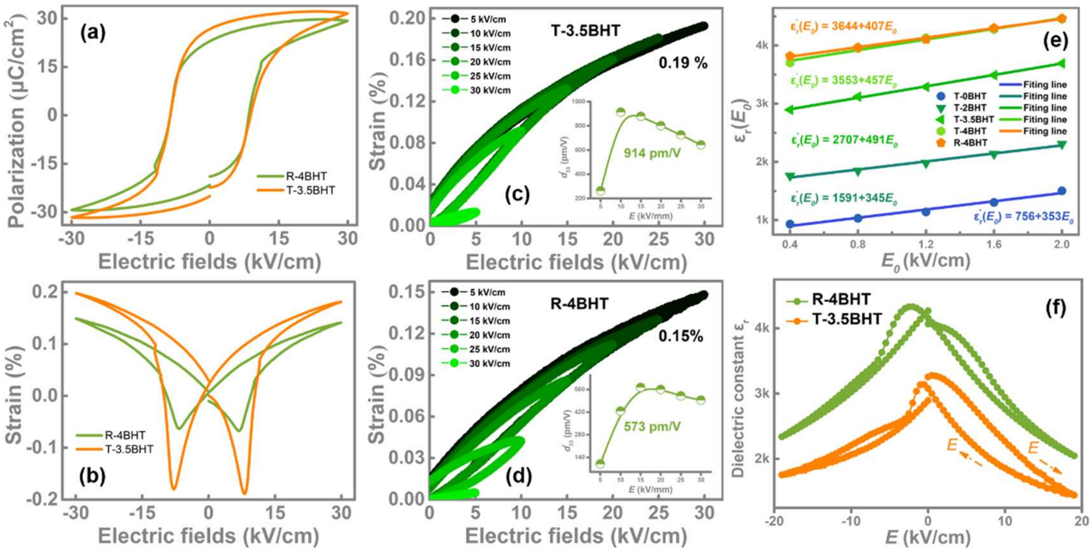

**Figure S10. Ferro-/piezoelectricity and the corresponding intrinsic or extrinsic contributions.** (a) *P*-*E* hysteresis loops for R-4BHT and T-3.5BHT. (b) Bipolar strain-electric field (*S*-*E*) curves for R-4BHT and T-3.5BHT. Unipolar strain-electric field (*S*-*E*) curves under different electric fields and corresponding calculated piezoelectric strain coefficient $d_{33}^*$ (**S/E**) for T-3.5BHT (c) and R-4BHT (d). (e) AC electric field dependent $\varepsilon_r$. (f) In-situ electric field dielectric curves.

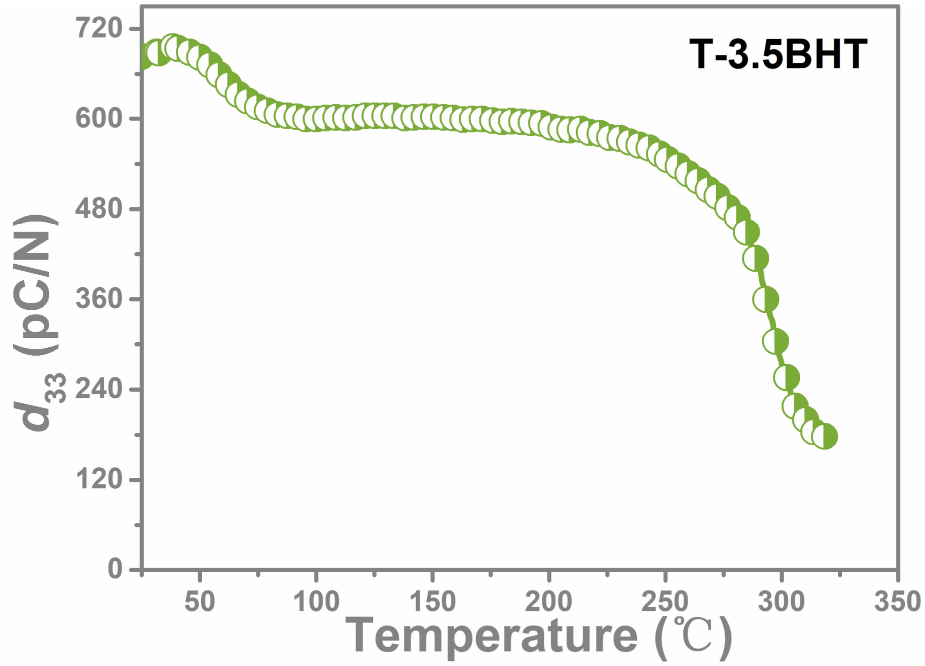


**Figure S11. Temperature fatigue resistance of piezoelectricity.** In situ temperature-dependent $d_{33}$ value of the T-3.5BHT ceramics.

Since the NPB phase boundary belongs to the temperature-dependent phase boundary,[3] the phase structure of the T-3.5BHT ceramics gradually deviates from the phase boundary with the increase of the temperature. The $d_{33}$ value of the piezoceramics is closely related to the phase structure, so the $d_{33}$ value of T-3.5BHT ceramics decreases firstly with increasing temperature due to the deviation of the phase boundary, then stabilizes after 100 °C, and finally decreases sharply due to the generation of the paraelectric phase near the Curie temperature of 260 °C.

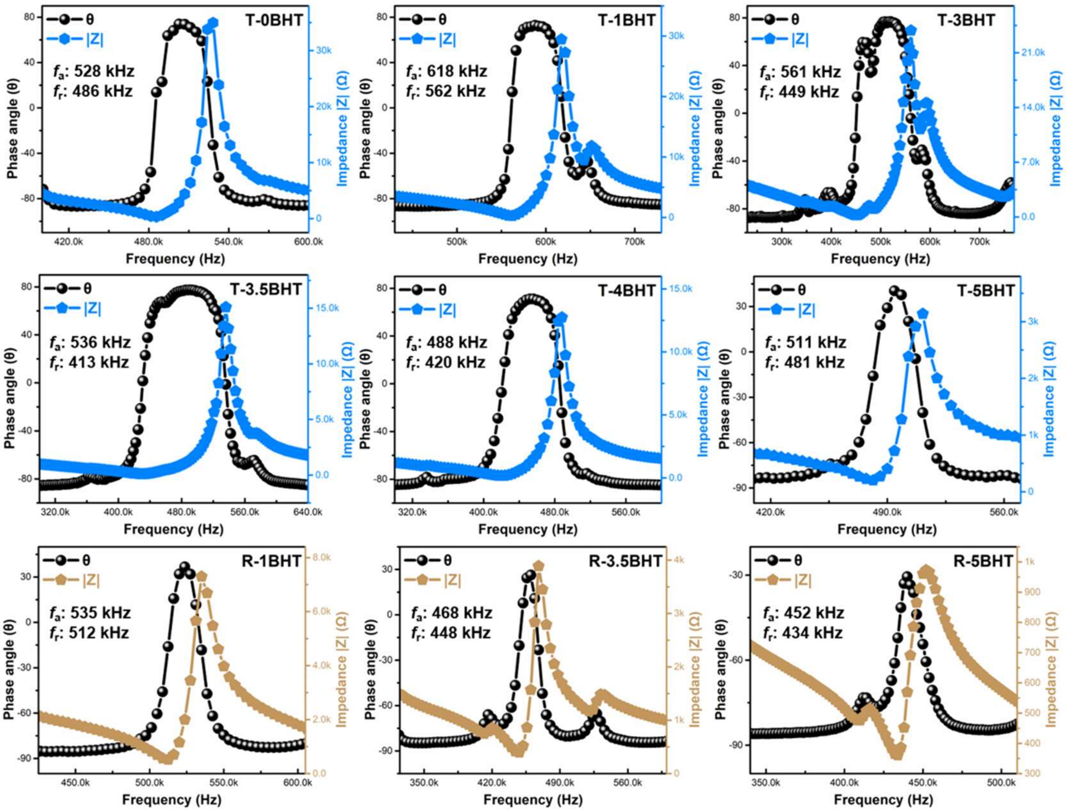


**Figure S12. Electromechanical coupling characteristics.** Impedance Z and phase angle θ against frequency of the *x*BHT ceramics measured at room temperature, where $f_r$ and $f_a$ are represented as resonance and antiresonance frequencies, respectively.

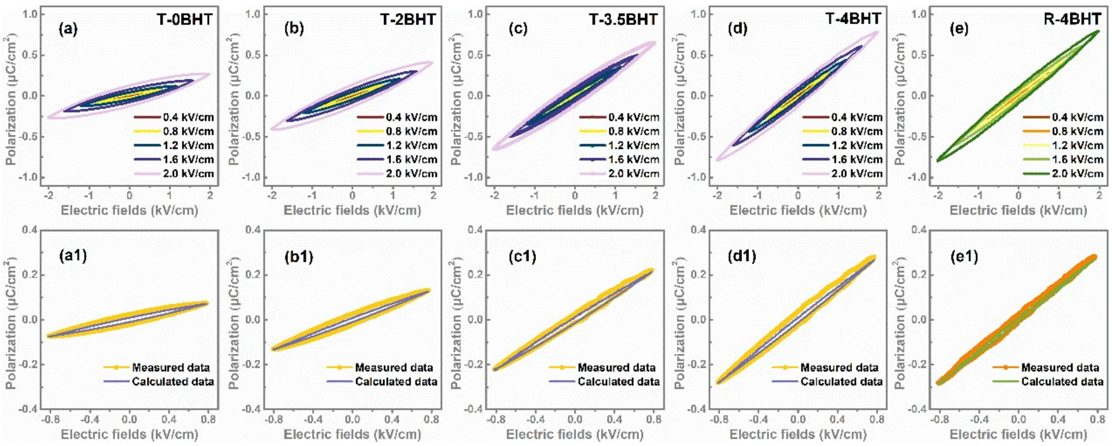


**Figure S13. The intrinsic or extrinsic contributions of piezoelectric and dielectric properties.** (a-e) *P-E* hysteresis loops under different electric fields for T-*x*BHT and R-4BHT ceramics. (a1-e1) The curves of measured and calculated *P-E* hysteresis loops at $E_0$ of 0.8 kV/cm for T-*x*BHT and R-4BHT ceramics.

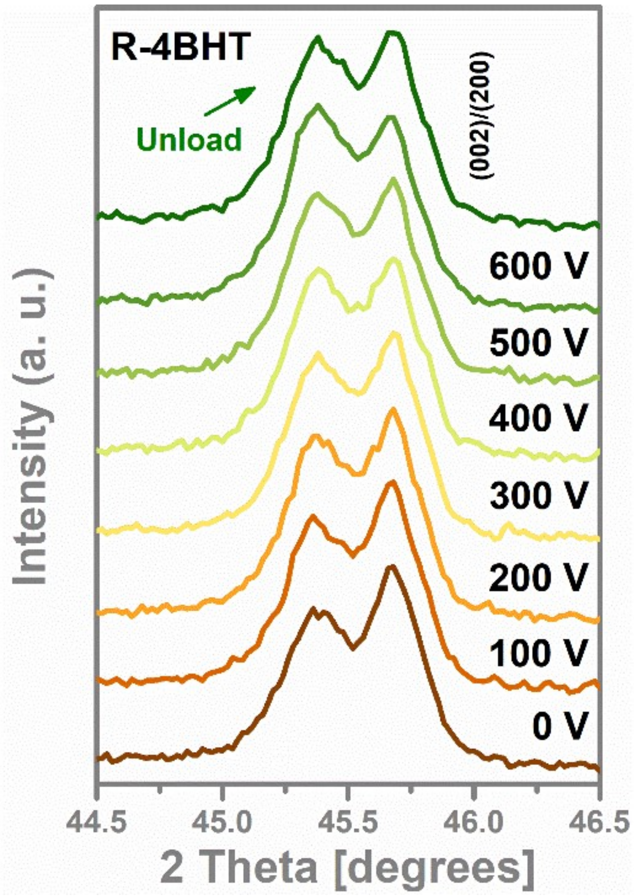


**Figure S14. Electric field-dependent XRD.** In-situ XRD versus the applied electric field for the R-4BHT ceramics.

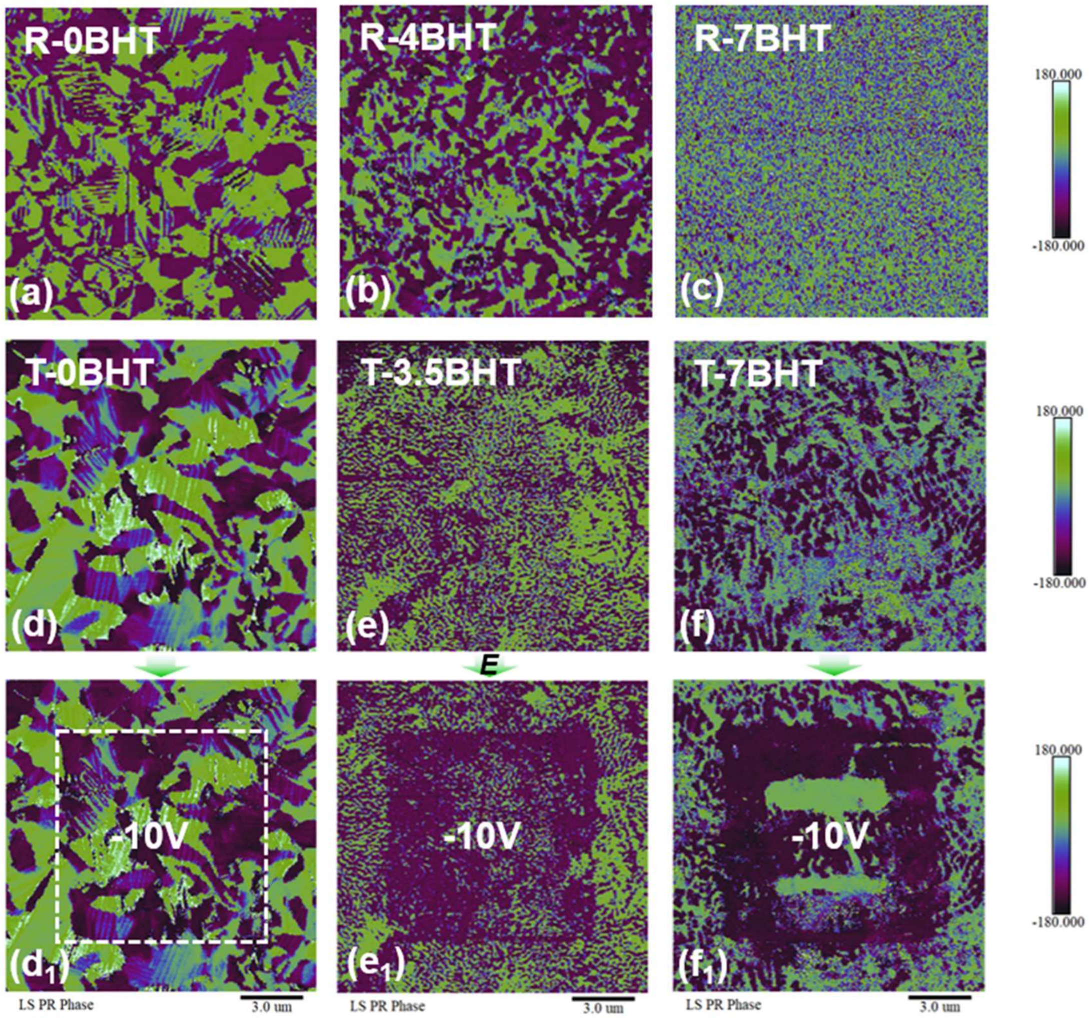


**Figure S15. Optimized vertical piezoresponse force microscopy (OV-PFM).** OV-PFM images of the R-*x*BHT ceramics for (a) $x = 0$, (b) $x = 4$ and (c) $x = 7$. OV-PFM images of the T-*x*BHT ceramics before (d-f) and after ($d_1$-$f_1$) applying -10 V DC tip biases.

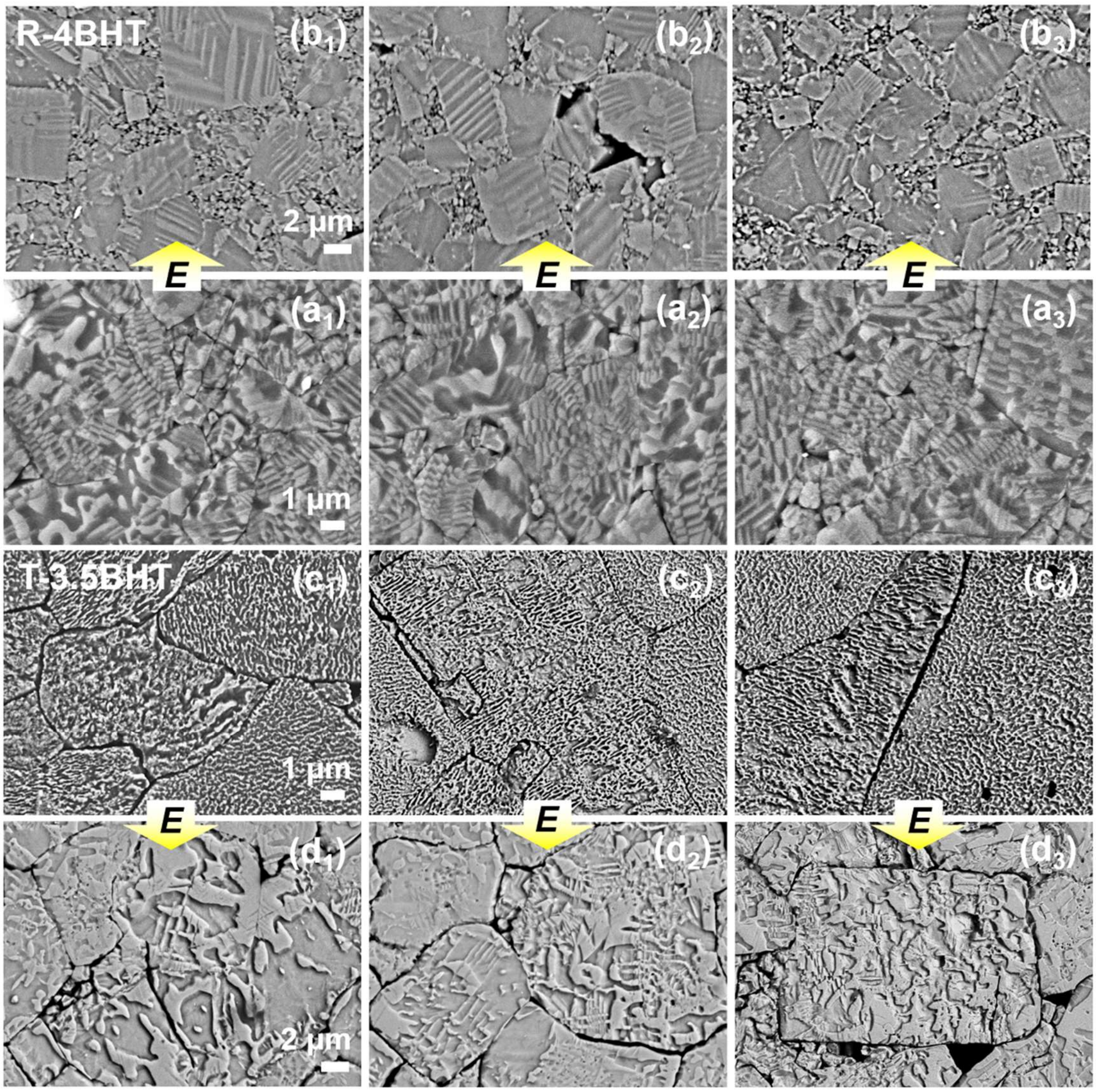


**Figure S16. The morphology of acid-etched domain.** SEM images of acid-etched domain of the R-4BHT ceramics before ($a_1$-$a_3$) and after ($b_1$-$b_3$) poled. SEM images of acid-etched domain of the T-3.5BHT ceramics before ($c_1$-$c_3$) and after ($d_1$-$d_3$) poled.

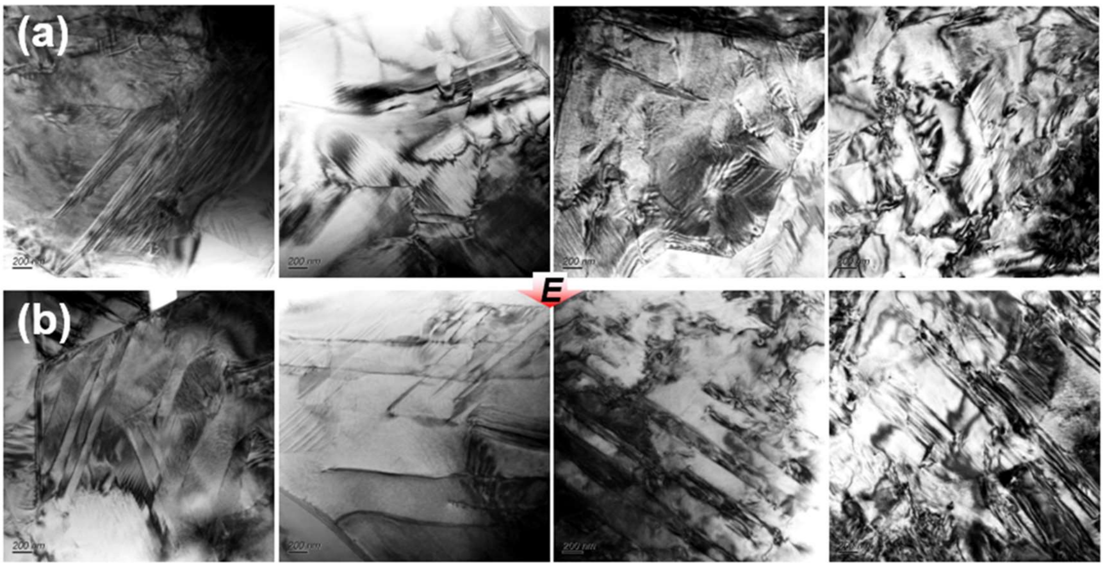


**Figure S17. Analysis of domain structure via transmission electron microscopy (TEM).** TEM images of the T-3.5BHT ceramics before (a) and after (b) poled.

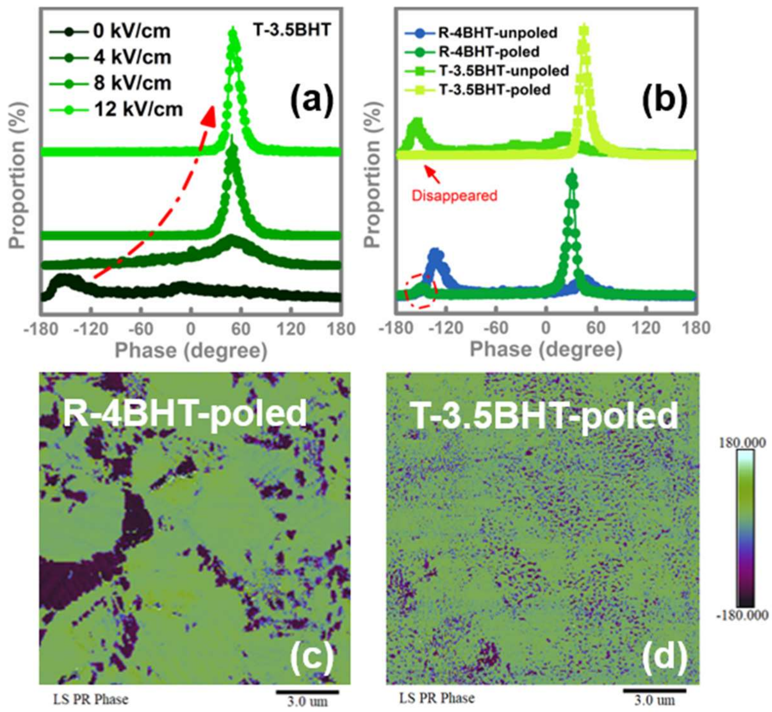


**Figure S18. Analysis of domain structure after poled.** (a) Corresponding phase proportion of domains for in-situ electric field OV-PFM images for the T-3.5BHT ceramics. (b) Corresponding phase proportion of domains for the R-4BHT and T-3.5BHT ceramics before and after poled. OV-PFM images for the poled R-4BHT (c) and T-3.5BHT (d) ceramics.

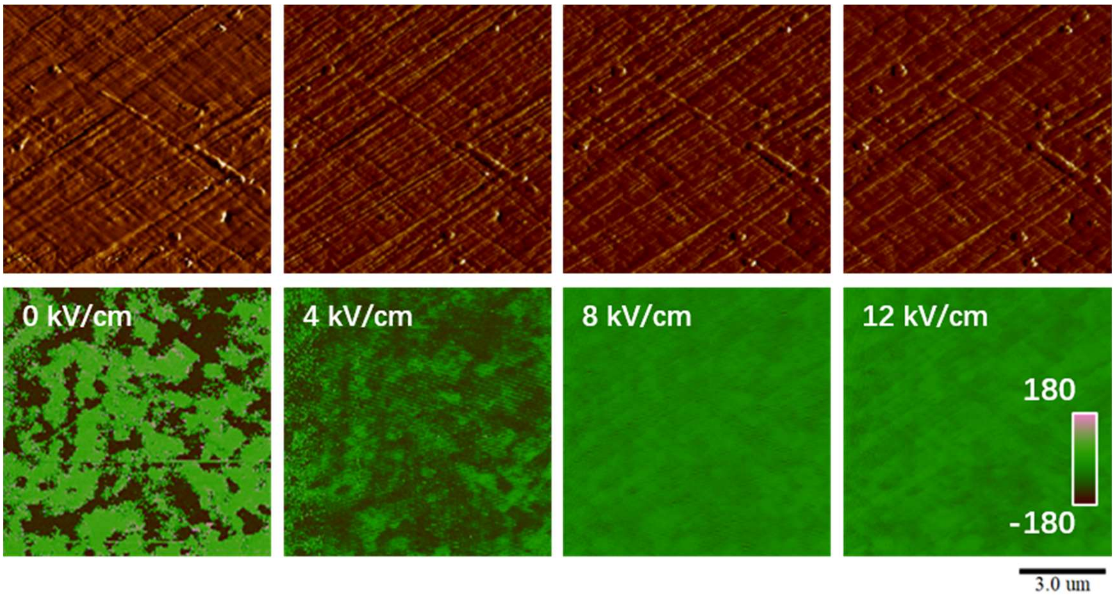


**Figure S19. Analysis of Field-induced domain evolution.** In-situ electric field vertical piezoresponse force microscopy (V-PFM) images for the T-3.5BHT ceramics.

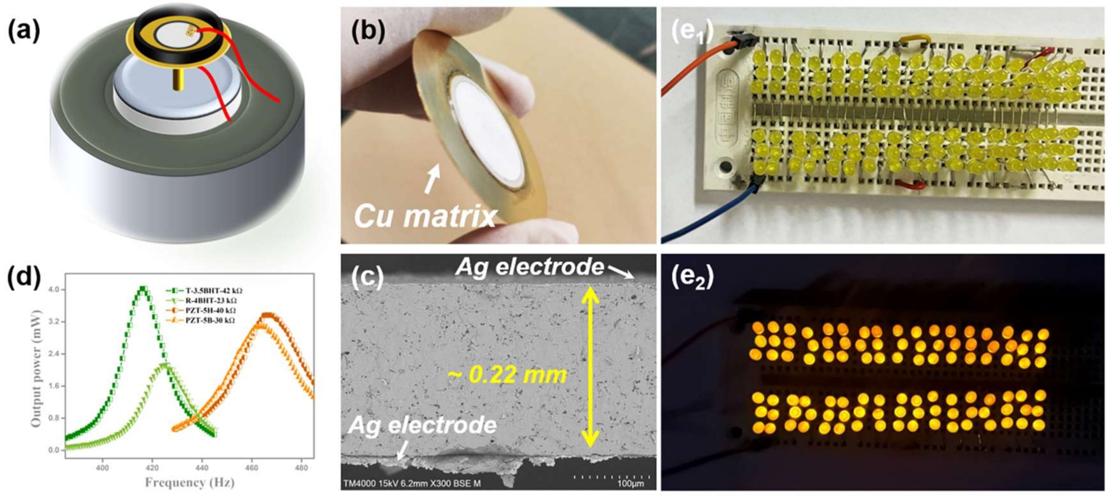


**Figure S20. PCD piezoelectric energy harvester.** (a) Schematic diagram of PCD piezoelectric energy harvester. (b) Cu matrix of the PCD piezoelectric energy harvester. (c) The cross-sectional SEM images of the T-3.5BHT ceramics in the prepared energy harvesters. (d) The output power comparison of the T-3.5BHT PCD PEH with other lead-based piezoelectric harvesters. (e) The real time lighting photo of light emitting diodes (LEDs) driven by the T-3.5BHT PCD PEH.

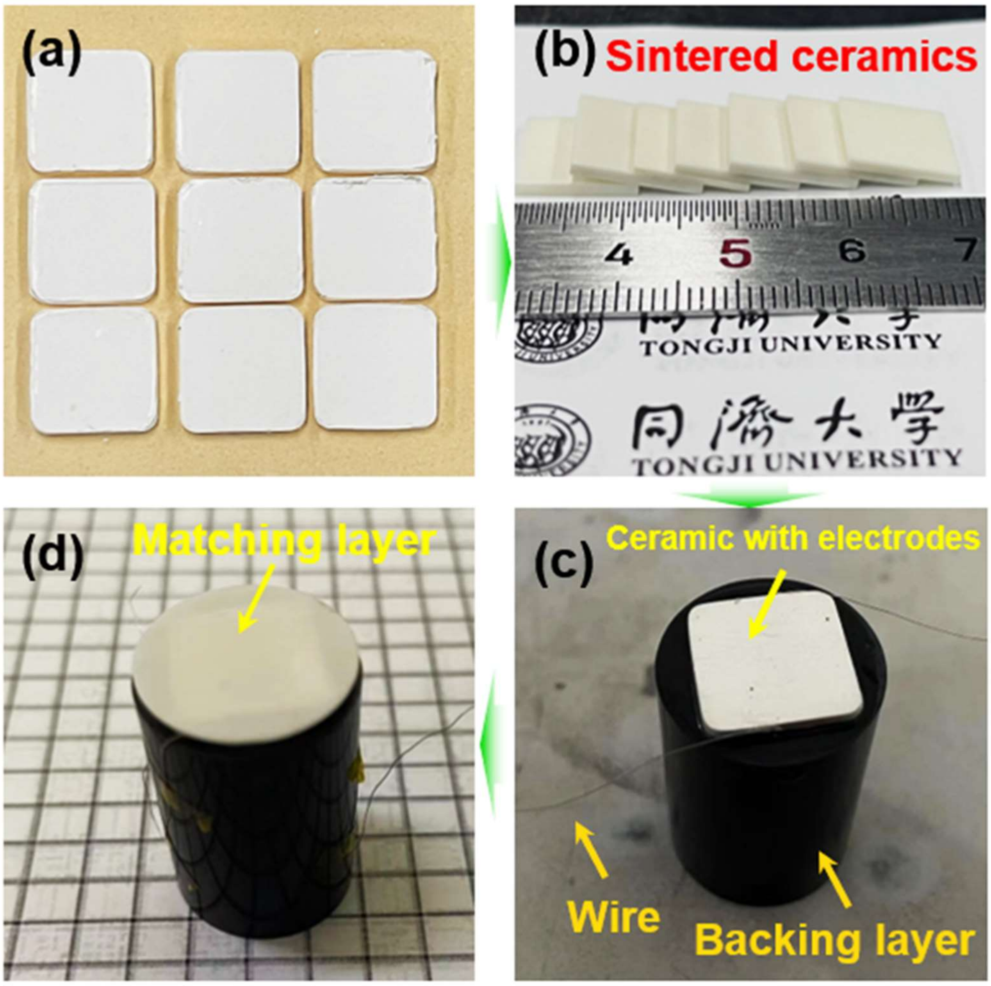


**Figure S21. Preparation process of the T-3.5BHT ceramic transducer.** (a) The green body of the T-3.5BHT ceramics before burn out the binder. (b) The T-3.5BHT ceramics after sintered. (c, d) The corresponding details of the backing layer, wire, ceramic with electrodes and matching layer for the T-3.5BHT ceramic transducer.

**Supplementary References:**


1. Gao, R. Shi, X. Wang, J. Zhang, G. & Huang, H. Designed Giant Room-Temperature Electrocaloric Effects in Metal-Free Organic Perovskite [MDABCO]($NH_4$)$I_3$ by Phase-Field Simulations. *Adv. Funct. Mater.* **31**, 2104393 (2021).
2. Pohlmann, H. Wang, J. J. Wang, B. & Chen, L. Q. A Thermodynamic Potential and The Temperature-Composition Phase Diagram for Single-Crystalline $K_{1-x}Na_xNbO_3$ ($0 \leq x \leq 0.5$). *Appl. Phys. Lett.* **110**, 102906 (2017).
3. Lv, X. Zhu, J. G. Xiao, D. Q. Zhang, X. X. & Wu, J. G. Emerging New Phase Boundary in Potassium Sodium-Niobate Based Ceramics. *Chem. Soc. Rev.* **49**, 671-707 (2020).
4. Wu, J. G. Xiao, D. Q. & Zhu, J. G. Potassium-Sodium Niobate Lead-Free Piezoelectric Materials: Past, Present, and Future of Phase Boundaries. *Chem. Rev.* **115**, 2559-2595 (2015)
5. Bian, L. et al. High-Performance [001]c-Textured PNN-PZT Relaxor Ferroelectric Ceramics for Electromechanical Coupling Devices. *Adv. Funct. Mater.* **30**, 2001846, (2020).
