# Supplementary material for: Multiscale reconfiguration induced highly saturated poling in lead-free piezoceramics for giant energy conversion
Source: Nat Commun. 2024 Mar 22;15:2560. doi: 10.1038/s41467-024-46894-5 (PMC10959963; doi:10.1038/s41467-024-46894-5)
Supplement: Supplementary file 3 — Description of Additional Supplementary Files [file 41467_2024_46894_MOESM3_ESM.pdf]

Description of Additional Supplementary Files

File Name: **Supplementary Movie 1**

Description: **Operation demonstration of the T-3.5BHT piezoelectric circular diaphragm (PCD) energy harvester.** By controlling the on or off of the vibrator, the T-3.5BHT PCD energy harvester can effectively turn on or off the connected light-emitting diodes (LEDs) using the designed circuit.
